# Workplace support, wellbeing and intention to leave among lone working healthcare assistants providing palliative and end-of-life care in the community: A mixed methods study

**DOI:** 10.1177/02692163251395576

**Published:** 2025-12-06

**Authors:** Katarzyna A. Patynowska, Emma Maun, Erin Raquel Fantoni, Tracey McConnell, Anne Finucane, Jonathan Clemo, Epiphany Leone, Natasha Wynne, Colette McAtamney, Felicity Hasson

**Affiliations:** 1 Marie Curie, Belfast, UK; 2 Institute of Nursing and Health Research, Ulster University, Belfast, UK; 3 Marie Curie, London, UK; 4 Marie Curie Palliative Care Research Department, University College London, London, UK; 5 School of Nursing and Midwifery, Queen’s University Belfast, Medical Biology Centre, Belfast, UK; 6 Clinical Psychology, Health in Social Science, University of Edinburgh, Edinburgh, UK; 7 Marie Curie, Edinburgh, UK

**Keywords:** palliative care, nursing assistants, health services research, occupational stress, psychosocial support, personnel turnover

## Abstract

**Background:**

Healthcare assistants play a critical role in providing palliative and end-of-life care in the community across many healthcare systems internationally. Despite working alone in emotionally demanding and unpredictable settings, no research has examined their psychological wellbeing or factors influencing turnover.

**Aim:**

To investigate the correlations between mental wellbeing, intention to leave their role and workplace support in lone working healthcare assistants providing palliative and end-of-life care in the community.

**Design:**

Explanatory sequential mixed methods study, using cross-sectional survey (Warwick-Edinburgh Mental Well-being Scale and Turnover Intention Scale, project team-developed questions), followed by interviews. Quantitative data analysed using descriptive and inferential statistics, qualitative data analysed using framework approach.

**Setting/participants:**

Lone working healthcare assistants from a UK non-profit organisation providing palliative and end-of-life care.

**Results:**

Among 218 survey respondents (22.5% response rate), 80% (*n* = 174) reported average to high mental wellbeing (mean = 52.2, SD = 8.6). Intention to leave was low (mean = 14.9, SD = 5.3). Higher wellbeing correlated with lower intention to leave (*r*(216) = −0.25, *p* < 0.001). Interviews (*n* = 14) and survey data revealed support from known individuals, particularly line managers, was most valued and accessed by 87.2% of respondents, significantly associated with higher wellbeing and lower turnover intention. Clinical supervision and peer support were frequently accessed and valued, though impact on wellbeing and retention varied. Anonymous online support services remained largely unused despite high awareness.

**Conclusions:**

The study challenges assumptions about psychological distress experienced by this workforce while showing that targeted, personalised workplace support strategies could be key to retention, offering evidence-based pathways for strengthening workforce sustainability.

**What is already known about the topic?**

- Healthcare assistants are one of the key patient-facing workers across various healthcare systems internationally, including palliative and end-of-life care in the community.
- The complexity of the healthcare assistant role is often overlooked, highlighting the need for appropriate education and support.
- The healthcare assistant role within palliative care, often requires them to be lone workers providing significant emotional labour which has a high potential for stress.

**What this paper adds?**

- To the best of the authors’ knowledge, this is the first paper to study the mental wellbeing of healthcare assistants providing palliative and end-of-life care in the community showing average to high wellbeing among this workforce.
- Greater mental wellbeing is associated with lower intention to leave the role among lone working healthcare assistants.
- Lone working healthcare assistants mainly access contact-based support from known persons, including line manager and team members, although opportunities to access support from peers were often lacking.

**Implications for practice, theory or policy**

- Service providers may consider exploring strategies to ensure lone working healthcare assistants can establish both professional and personal connections within the workplace, as inadequate support may adversely affect staff retention.
- Raising awareness of and increasing access to a range of support mechanisms is needed to meet the needs of lone working healthcare assistants, to avoid relying on a single support source such as a line manager.
- Further research may explore the specific support mechanisms and contextual factors that contribute to the wellbeing of healthcare assistants providing palliative and end-of-life care in the community.

## Background

The global demand for palliative care is rising due to ageing populations, increased prevalence of chronic diseases, with a shift towards community-based delivery of end-of-life care.^1–5^ This growing need coincides with healthcare shortages worldwide, leading to an increasing reliance on support staff across all clinical settings.^6–8^ These support workers, known by various titles internationally including healthcare assistants (term used in this study), certified nurse aids, personal care assistants or unregulated care providers, represent a diverse but essential workforce with significant variation in training, regulation and scope of practice across jurisdictions.^9,10^

In community-based palliative and end-of-life care, healthcare assistants are key patient-facing workers who commonly work alone to provide personal care, emotional support and assistance with daily living activities to patients and their informal caregivers.^11–14^ Their responsibilities extend beyond basic care to include holistic care provision, ongoing assessments and escalation of concerns to other healthcare professionals.^14–17^ Despite their crucial role in sustaining community-based palliative and end-of-life care services, healthcare assistants remain undervalued and their contributions are often overlooked.^11,18^

A defining characteristic of palliative and end-of-life care in the community is the prevalence of lone working, where healthcare assistants work independently with limited direct supervision and isolated from peers.^9,13,19–22^ Lone working is recognised as inherently complex due to the unpredictability of the risks, decision-making, challenges that workers may face and limited immediate professional support when challenges arise.^23^ Research indicates that lone workers face increased risk of poor health status compared to non-lone workers.^24^ In community-based palliative and end-of-life care lone working amplifies these challenges for healthcare assistants due to exposure to death and dying, intense emotional labour,^25–31^ isolation and loneliness,^16,31,32^ inadequate training and preparation for the role^13,16,31,33–36^ and unpredictable environments and family situations.^16,37^

The psychological wellbeing of healthcare workers has been identified as an important factor influencing turnover intentions^38^ and quality of care provided.^39–41^ While wellbeing among nurses, doctors and other regulated healthcare professionals has received substantial research attention,^38,42–44^ equivalent evidence for healthcare assistants remains limited. This gap is particularly concerning given that healthcare assistants often work in challenging conditions with little institutional support.^45,46^

Despite healthcare assistants’ important role in community-based palliative and end-of-life care delivery and their exposure to significant occupational stressors through lone working arrangements, no research has examined the relationships between their psychological wellbeing, intention to leave their roles, and workplace support factors, such as awareness of, access to, and helpfulness of various support forms. This includes support from known individuals, and anonymous, online, self-directed support (see Table 3 for a full list and Box 1 for definitions). Such a knowledge gap represents a critical barrier to developing effective workforce retention strategies and ensuring sustainable care services. This study addresses this gap by describing levels of mental wellbeing and intention to leave and investigating how workplace support relates to wellbeing and turnover intentions among lone working healthcare assistants providing palliative and end-of-life care in the community.

Box 1. **Definitions of workplace support forms**

**Clinical supervision** – a formal process of professional support and learning which enables individual practitioners to develop knowledge and competence, assume responsibility for their own practice and enhance service user protection and safety of care in complex situations.^57^ The framework used within the recruiting organisation is Resilience-based Clinical Supervision.^58^

**Employee Assistance Program** – free, confidential and independent programme including a variety of support for staff mental and emotional wellbeing such as free 24/7 Telephone Hotline offering expert counselling to support staff emotional and mental wellbeing and expert advisors for practical legal information or debt service.^59^

**Health and Wellbeing Hub** – internal organisational website containing variety of internal and external resources supporting mental, physical, spiritual and financial wellbeing, links to groups, events and mental health first aiders.^59^

**Unmind app** – digital platform which gives staff access to scientifically backed tools created by experts in neuroscience, cognitive behavioural therapy, mindfulness and positive psychology.^59^

**Schwartz Rounds** – Schwartz Rounds provide a structured forum where all staff, clinical and non-clinical, come together regularly to discuss the emotional and social aspects of working in healthcare. The purpose of Rounds is to explore the challenges and rewards that are intrinsic to providing care, not to solve problems or to focus on the clinical aspects of patient care.^60^

## Methods

### Design

We adopted a two-phase mixed-method explanatory sequential design^47^ including an online cross-sectional survey and qualitative semi-structured interviews. This approach was chosen to leverage the strengths of quantitative and qualitative approaches. Validated scales provided robust prevalence and correlation data, while interviews contextualised these findings. The study was reported following the GRAMMS guidelines.^48^

### Setting

Participants were recruited from a non-profit organisation that supports over 60,000 terminally ill patients annually across the UK, with healthcare assistants providing planned and unplanned palliative and end-of-life care in the community, mainly as overnight 1:1 care.

### Participants

Participants were healthcare assistants, who worked alone in patients’ homes for any amount of time in their job. At the start of the study (01/05/23), 969 lone working healthcare assistants were employed in services providing palliative and end-of-life care in the community.

### Recruitment

We adopted convenience sampling to recruit by inviting all eligible staff to participate. Consent was implied through anonymous survey submission. At the end of the survey, participants could express interest in an online interview by completing a separate form. Interested individuals were contacted by KP via telephone or email to gain consent and schedule interviews. A prize draw for gift vouchers was used to encourage survey responses and all interview participants received a voucher as a token of appreciation.

### Questionnaire and measures

The questionnaire included the following (Supplemental Material):

*Wellbeing* was assessed using the Warwick-Edinburgh Mental Well-being Scale,^49,50^ a validated scale with 14 items.^50,51^ Overall score (range 14–70) was calculated by summing item scores, higher scores indicating higher level of mental wellbeing.^52^ Cut-offs were calculated for average wellbeing (within 1 standard deviation of the mean, scores 44–60), high wellbeing (scores 61–70) and low wellbeing (scores 14–43).^52^

*Intention to leave* was assessed using the six-item Turnover Intention Scale (TIS-6).^53,54^ The scale has been validated for measuring turnover intention and predicting actual turnover in the communications sector with acceptable reliability, Cronbach’s alpha, 0.80 and used in health sector studies.^53,55^ A five-point Likert scale from “never” (1) to “always” (5) was used to rate responses to questions such as “*how often have you considered leaving your job*?.” The midpoint of the scale is 18 (range 6–30), with higher scores indicating a greater intention to leave. Scale scores were used (range 6–30), with higher scores indicating a greater intention to leave. A score of 18 or above can be interpreted as indicating an intention to leave the organisation.^56^

Reliability and validity of both wellbeing and intention to leave measures were acceptable in the sample (Supplemental Material).

*Current workplace wellbeing support* was assessed by asking about participant awareness of, access to, and opinions of helpfulness relating to a list of forms of support, including support from a line manager and clinical supervision (see Box 1 for definitions). Participants were asked if they were aware of, and if they had accessed each support option. Those who had accessed a form of support were asked about the helpfulness of the support, rating it from “not at all helpful” (1) to “very helpful” (4).

*Open-text questions* explored participants’ reasons for intending to stay/leave their roles and their views on how the organisation could improve healthcare assistants’ retention.

### Data collection

The survey (Supplemental Material) was available online via Microsoft Forms 12/04/23–7/06/23. All participants who completed an expression of interest form at the end of the survey (*n* = 14) took part in the online interviews (for interview guide please see Supplemental Material) conducted by KP between 19/07/23 and 31/08/23.

### Data analysis

#### Quantitative data

Data analysis was undertaken by RF and EM. Pearson correlation coefficients and ANOVA were used to assess the relationship between wellbeing and intention to leave and Spearman and bi-point serial correlation coefficients were calculated for analysis of correlations between other variables and wellbeing and intention to leave. Analyses were performed using SPSS version 29.0.1.0 and R.

#### Qualitative data

Free-text survey data were managed using Microsoft Excel. Interviews were transcribed verbatim. Anonymised transcripts were thoroughly reviewed multiple times to familiarise with the data, and reflexive notes were maintained.^61^ Both data sets were printed and combined for further analysis. We employed an analytical framework^62^ to focus our analysis on the key areas of interest within the wider dataset, while examining predetermined themes alongside emergent findings. Our analytical approach was guided by a pragmatic approach including the following: immersion in the data, coding, developing an analytical framework, applying the framework, the establishment of categories and themes and refining the framework.^62–64^ This inductive and deductive process was conducted independently by KP and FH, with collaborative refinement of the themes to ensure accuracy and coherence.

#### Integration

The survey data guided the development of the interview schedule (Supplemental Material). Results from both phases were analysed concurrently and integrated using “following a thread technique”^65^ by exploring specific themes across data sets to create a coherent narrative. The findings were analysed through a collaborative and iterative approach among the research team and advisory group members.

*Techniques* to enhance objectivity and depth of analysis included second coder, negative case seeking/analysis, participant feedback (four responses received from interview participants) and triangulation.^66^ Comparing and combining the analysis between two researchers provided additional analytic insights and reduced errors and threats to validity.^67–69^ Discussion with the research team and advisory group helped to ensure the relevance, credibility and trustworthiness of identified themes.

## Results

### Overview

In total, 218 healthcare assistants completed the surveys (response rate 22.5%). Complete data were available for the main variables of interest: wellbeing and intention to leave, therefore cases with missing data on sociodemographic and work variables were retained and variation in *N* reported in subsequent tables. We conducted 14 online interviews ranging between 24 and 67 min (mean 41 min). Participant characteristics are described in Table 1. Pseudonyms were assigned to all participants to protect their anonymity in the presented quotations.

**Table 1.** Sample characteristics.

Variable	Category	Survey participants, *N* = 218		Interview participants, *N* = 14	
		Frequency^a^	% Of valid responses/mean (SD)	Frequency	% Of valid responses/mean (SD)
Gender^b^	Female	202	92.7	14	100
	Male	9	4.1	-	
Age	Mean (SD)	200	Mean 51.3 (SD 11.0)	14	Mean 51.2 (SD 13.9)
Ethnicity^c^	White	181	85.0	14	100
	Ethnic minorities	32	15.0	-	-
Proportion of time spent lone working	All/almost all	165	75.7	14	100
	Most	23	10.6	-	-
	About half	7	3.2	-	-
	Some	16	7.3	-	-
	Almost none	7	3.2	-	-
Contract	Contracted hours	169	77.9	10	71
	Bank (As and when required)	48	22.1	4	29
Type of hours	Full time	83	39.3	2	14
	Part time	128	60.7	12	86
Place of work	England	144	68.6	8	57
	Northern Ireland	18	8.6	2	14
	Scotland	39	18.6	4	29
	Wales	9	4.3	-	-
Total HCA experience^d^		199	Mean 18.7 (SD 11.7)	14	Mean 14.4 (SD 12.0)

SD: standard deviation.

a Missing data: gender (seven participants, 3.2%), age (18, 8.2%), ethnicity (5, 2.3%), type of hours (7, 3.2%), place of work (8, 3.7%), years of experience at HCA in current employing organisation and total (19, 8.7%), overall level of missingness 10.6%.

b Includes survey responses: Prefer not to say (4), Prefer to self-describe (2) and missing (1).

c Ethnic minorities included healthcare assistants self-reporting as Asian (3), Black (23) and other/mixed categories (6).

d Missing includes survey participants whose years of experience exceeded their age or required commencing work before age 16.

Of the survey participants, 163 (74.8%) had previous experience in health and care settings. All interview participants and 184 survey participants (84%) worked in the planned service, where they most often provided 1:1 care for a one patient/family overnight.

### Wellbeing and intention to leave

Most healthcare assistants reported average to high levels of wellbeing with a mean score of 52.2 (SD = 8.6) and a mean intention to leave score of 14.9 (SD = 5.3; Table 2). We found a negative correlation between healthcare assistant wellbeing and intention to leave, indicating that healthcare assistants with higher levels of wellbeing were less likely to express an intention to leave (*r*(216) = −0.25, *p* < 0.001). When grouped according to statistical cut-offs, healthcare assistants with high wellbeing had lower mean intention to leave than those categorised as having low wellbeing (ANOVA, *F* statistic (2) = 3.8, *p* = 0.02 and post-hoc Tukey test, *p* = 0.02 for relevant groups, Table 2). No significant correlations were found between wellbeing, intention to leave and other characteristics of the sample, including age, time spent lone working and years of experience, so testing of the relationship between wellbeing and intention to leave was not adjusted for other variables (results of correlations in Supplemental Material).

**Table 2.** Wellbeing mean and statistical cut-offs, with intention to leave scores.

Category	Frequency	Wellbeing score (range 14–70), mean (SD)	Wellbeing score % of responses	Intention to leave score (range 6–30), mean (SD)
Sample mean score (SD)	218	52.2 (8.6)		14.9 (5.3)^a^
Wellbeing statistical cut-offs^b^				
High wellbeing (score 61–70)	36	65.1 (2.7)	16.5%	13.1 (5.1)
Average (±1 SD, score 44–60)	138	52.7 (4.4)	63.3%	14.8 (5.2)
Low wellbeing (score 14–43)	44	39.7 (3.3)	20.2%	16.3 (5.3)

SD: standard deviation.

a Pearson correlation (Wellbeing and Intention to leave), *r*(216) = −0.25, *p* < 0.001.

b Statistical cut-offs for high and low wellbeing estimated following WEMWBS guidance (https://warwick.ac.uk/fac/sci/med/research/platform/wemwbs/using/howto/).

### Workplace support

Participants demonstrated a high level of awareness and access to contact-based support from people known to them. About 97.2% had accessed support provided by either line managers, clinical supervisors, family and friends, other healthcare assistants or other healthcare workers (Table 3). Anonymous and online self-directed forms of support were less well known and were accessed by 17.4% or fewer participants (Table 3). Where participants accessed any form of support, over 90% found it moderately helpful or very helpful, with no statistically significant differences by support type (Supplemental Material).

**Table 3.** Participant awareness and accessing of different types of support: *N*, % and correlations with wellbeing and intention to leave scores.

Type of support	Aware of support	Accessed support	Correlation (*p*-value) between accessed support and	
	*N* (%)^a^	*N* (%)^a^	Wellbeing^b^	Intention to leave^b^
Support from known persons	218 (100)	212 (97.2)		
Line manager	215 (98.6)	190 (87.2)	0.30 (<0.001)	−0.27 (<0.001)
Clinical supervision	213 (97.7)	166 (76.2)	0.03 (0.662)	0.02 (0.733)
Family and friends	211 (96.8)	121 (55.5)	0.04 (0.575)	0.00 (0.964)
Other healthcare assistants	205 (94.0)	157 (72.0)	0.10 (0.156)	−0.09 (0.199)
Other healthcare workers	198 (90.8)	149 (68.3)	0.11 (0.115)	−0.10 (0.158)
Anonymous and online self-directed support	202 (92.7)	38 (17.4)		
Health and wellbeing hub	188 (86.2)	19 (8.7)	0.08 (0.231)	−0.06 (0.382)
Employee assistance program	145 (66.5)	21 (9.6)	0.09 (0.195)	−0.02 (0.732)
Unmind app	78 (35.8)	7 (3.2)	-	-
Schwartz rounds	53 (24.3)	4 (1.8)	-	-

SD: standard deviation.

a Percentage of sample of 218 participants.

b Bi-point serial correlations presented only for those variables with more than 10 cases.

### Line manager

Accessing line manager’s support was associated with higher wellbeing (*r*(216) = 0.30, *p* < 0.001, Table 3) and lower intention to leave (*r*(216) = −0.27, *p* < 0.001, Table 3). Line managers serve as the primary point of contact for addressing the diverse support needs of lone working healthcare assistants, including clinical queries, training needs, emotional support and operational management.

I think most people probably go to their line managers first and foremost, because they are very much considered your first point of contact for anything that you need within your job. (Harper)

Line managers are the main point of contact for urgent emotional support and debrief after a difficult shift.

I came across a situation and as soon as I got home the next morning, I emailed and then asked if a senior nurse or somebody could ring me later in the afternoon, once I was wakened, just to talk through the situation that had happened. That was really, really useful, just a chance to debrief almost. Just talk through a really difficult situation. (Lisa).

To fulfil their role as a first point of contact, line managers need to be available to provide a timely response to staff concerns and questions.

We were emailing them, and it was taking ages for them to come back to you. Or you were trying to phone, and nobody was answering you. Or maybe somebody needed annual leave put in and it wasn’t working on the system, and you’re trying to get them to approve it, and they weren’t coming back. So that caused a lot of stresses. (Chris)

Effective relationship with the manager is underscored by psychological safety and being seen as an individual.

They are really good managers, and they are really interested in you. They know all about you. You talk to them about any of the girls and they know everything about everybody. They really take an interest in you. They are very, very supportive. (Chris)

With my line manager, I feel very supported. And I feel very safe that if I had any concerns, or I wasn’t feeling a hundred percent, I know that I could go and talk to them and that the support would be there. (Sam)

### Clinical supervision

No association was found between accessing group clinical supervision and wellbeing (*r*(216) = 0.03, *p* *=* *0.662*, Table 3). However, increased helpfulness of clinical supervision was associated with increased wellbeing (*r*(164) = 0.39, *p* < 0.001, Supplemental Material) and lower intention to leave (*r*(164) = −0.32, *p* < 0.001, Supplemental Material). Qualitative data demonstrated that clinical supervision was viewed as valuable for professional development by providing a structured forum for emotional and social support from team members.

That’s where you go and you share different experiences and you almost workshop it as a group to try and figure out, OK, if this happens again, what should we do? What’s best practice? And things like that. (Harper)

Clinical supervision sessions offer reassurance by enabling staff to identify shared challenges and concerns, thereby reinforcing a sense of collective experience.

It just gives you assurance that everybody is going through the same problems as what I am, regarding patients with certain issues and what have you. And that I’m not the only *one* who’s not sure if I should have done it this way or that way. We all just help each other and guide each other. (Laura)

The group sessions, particularly in-person, provide lone workers with opportunities to connect with colleagues and foster stronger relationships within the team.

When you are on your own, you see all these names and you’ve no clue who these people are. So it’s really good to sit down with colleagues and have a cup of tea and chat and catch up with everybody and get to know people better. (Lisa)

### Support from other healthcare assistants

We did not find significant correlations between accessing support from other healthcare assistants and wellbeing (*r*(216) = 0.10, *p* = 0.156, Table 3) or intention to leave (*r*(216) = 0.09, *p* = 0.199, Table 3). However, for those accessing this support, increased helpfulness of this support was associated with increased wellbeing (*r*(155) = 0.22, *p* = 0.006, Supplemental Material). Qualitative data emphasised the importance and the need for peer support among lone working healthcare assistants to reduce loneliness.

Just somebody else to talk to. As I say, it’s the talking aspect. Somebody else to talk to about situations and how you’ve felt in a certain situation. And also, just having that contact. Just a contact that I can. . . sometimes it would be nice if, when I’m on a night shift and there was somebody else on night shift that maybe would message in the night. (Marie)

Lone workers may experience loneliness and isolation due to the nature of their role, which often involves prolonged periods without contact with other team members

Ten and a half weeks in between actually seeing another colleague and having a chat. But there’s not really much chance to speak with colleagues, to speak with other HCAs. (Andrea)

### Anonymous and online self-directed forms of support

Minority of participants (17%) accessed at least one form of online or anonymous support. Preference for anonymous support was indicated in certain situations, particularly for issues affecting their work that are not work-related.

I think maybe if there were issues, not so much with work, more on my emotional side at home. Maybe if I had issues with my home life, then maybe then I would use it. Looking back, maybe it would have been ideal to use, not this last year, but the years leading up to last year. (Val)

One of the reasons for a low access of anonymous and online self-directed forms of support may be low awareness of these forms of support.

I think it’s poorly advertised, first of all. We were told about it during our induction and then it was never mentioned again. So I expect people have maybe heard about it in passing and they’ve completely forgotten about it. (Harper)

## Discussion

This study is the first to examine mental wellbeing of lone working healthcare assistants providing palliative and end-of-life care in the community, demonstrating moderate to high levels of mental wellbeing among this workforce (mean WEMWBS score 52.2, SD = 8.6) and low intention to leave their roles (mean TIS-6 score 14.9, SD = 5.3). We found a negative correlation between wellbeing and turnover intention (*r* = −0.25, *p* < 0.001). Healthcare assistants predominantly accessed contact-based support from known individuals, with line manager support being most frequently utilised (87.2%) and associated with higher wellbeing and lower turnover intention. While clinical supervision was widely accessed (76.2%), its effectiveness varied, with perceived helpfulness correlating with improved outcomes. Anonymous and online support options were rarely used (17.4% or fewer), despite high awareness levels. A common feature of all forms of support identified in our study was the timely, individualised delivery mode, typically involving in-person, virtual, or phone-based interactions. While the reasons why healthcare assistants access different forms of support were beyond the scope of this study, previous research suggests healthcare assistants need social and emotional support from team members and managers to promote mental wellbeing.^18,70,71^

Participants reported wellbeing scores that appeared higher than some healthcare professionals across general settings,^42,72^ hospice settings^73^ and the UK population average.^50^ However, direct cross-study comparisons should be approached cautiously given potential differences in populations, contexts, timing of data collection, and healthcare settings across studies. Evidence suggests that healthcare workers’ mental wellbeing influences individuals, organisations and care quality.^38–41^ Palliative care professionals, such as nurses and doctors, often experience emotional exhaustion intensified by ethical demands.^74^ Some studies suggest burnout is less prevalent among hospice staff than in other healthcare settings, but evidence remains inconclusive.^75^ Despite the potential for compassion fatigue in caregiving roles,^76,77^ it is possible that the ability to provide high-quality holistic care in the community and building relationships with patients and families may act as protective factors.^41,78^ The predominantly 1:1 overnight care model in our sample may be protective compared to roles requiring multiple patient contacts throughout a shift, as it allows for deeper therapeutic relationships and may reduce stressors associated with frequent transitions between patients. Additionally, our study indicates that support mechanisms tailored to the specific needs of lone workers, such as line manager support and clinical supervision, appear to play a role in maintaining wellbeing, aligning with findings from other studies.^41,78^

Participants in this study confirmed the value of support due to the emotional demands of their role and the complexities of lone working. Our results show that the most frequently accessed form of support was contact with line managers. This is unsurprising, as the line manager is typically the primary point of contact, particularly given the challenges in fostering communication and connexion with colleagues across different roles. Accessing managers’ support was positively associated with higher wellbeing and lower intention to leave. Other studies have similarly highlighted the importance of managerial support for home care workers,^31,32^ hospice staff^79^ and generally for healthcare workers.^42^ However, this reliance on individual managers also presents potential risks. Overdependence on line managers creates vulnerabilities when managers are ineffective, experience burnout themselves, or leave their role. Organisations should therefore develop diversified support systems rather than relying solely on key individuals, ensuring access to multiple support forms that remain resilient to staff changes.

Despite the importance of line manager support, participants also sought informal support from peers to mitigate isolation and loneliness. However, accessing peer support proved challenging. Our quantitative findings showed no significant correlation between simply accessing peer support and wellbeing outcomes. Instead, the perceived helpfulness of peer support, rather than its availability, was associated with improved wellbeing. This distinction suggests that support quality matters more than access alone. Potential barriers to meaningful peer interaction may include geographical dispersion of lone workers, different shift patterns limiting opportunities for contact, and the need for structured facilitation to enable effective peer connections. This finding aligns with research indicating that talking to colleagues about emotionally impactful events is a common coping strategy among palliative care professionals^13,80,81^ who highlight the need for time to talk and the emotional safety of the team.^80^

While no direct correlations were found between accessing resilience-based clinical supervision and wellbeing or intention to leave, when clinical supervision was considered helpful, this intervention was correlated with higher wellbeing and lower intention to leave, a finding supported by our qualitative data. Previous research suggests that clinical supervision can support practitioners’ self-care,^81–83^ but not all practitioners find clinical supervision helpful.^81^ Varied international models of clinical supervision show mixed effects on nursing practice^84^ and its effectiveness depends on having a skilled facilitator to create a safe, trusting environment.^58,85^

Finally, our study found a negative relationship between mental wellbeing and intention to leave, consistent with other research linking burnout and emotional exhaustion to increased intention to leave among nurses^86^ and physicians.^87^ Studies of work-related stress and wellbeing among healthcare staff show that the provision of support resources and positive perceptions of teamwork can significantly reduce turnover intention.^86,88,89^ Future research should explore the specific mechanisms and contextual factors that contribute to the enhanced wellbeing observed among lone working healthcare assistants providing palliative and end-of-life care in the community. Theory-based approaches such as realist research may be valuable for understanding how, why, and in what circumstances peer support works for this population. Our cross-sectional findings suggest that targeted workplace support strategies may represent promising areas for intervention development, though longitudinal research is needed to establish causal relationships.

### Implications for practice

This study highlights the importance of supporting the wellbeing of healthcare assistants. As they are regularly exposed to death, suffering, and grief, it is vital to ensure they receive support to manage psychological distress they may experience. The findings from this study provide community-based palliative and end-of-life care providers with an evidence base to develop organisational practices that better support their staff.

### Strengths and limitations of the study

Strengths include the novel emphasis on lone working healthcare assistants, a demographic underrepresented in previous research, with a substantial sample size of 218 participants. The 22% survey response rate, while presenting potential for non-response bias, is comparable to other healthcare worker surveys involving shift workers and geographically dispersed populations (e.g. recent adult social care workforce survey).^90^ We cannot determine how non-responders might differ from responders regarding wellbeing or turnover intention, which represents a limitation affecting generalisability. Despite this limitation, the mixed-methods design allowed us to explore findings in depth with interview participants from this understudied population. Additionally, we acknowledge that our sample demographics (92.7% female, 85% White) may limit generalisability to more diverse healthcare assistant populations globally. However, our sample was representative of the healthcare assistant workforce within the recruiting organisation. The study’s cross-sectional design did not allow the direction of the association between wellbeing and intention to leave to be explored. Future research could collect longitudinal data and include adjusted analyses to explore this further. A small number of respondents worked alone occasionally, representing 3% of the sample, so their inclusion likely had minimal impact on the findings

## Conclusion

This first study of lone working healthcare assistants’ mental wellbeing challenges assumptions about psychological distress in this workforce, showing that they experience moderate to high levels of mental wellbeing, which is negatively correlated with intention to leave their jobs. Contact-based support, particularly from line managers, is valued and shows positive associations with wellbeing outcomes. While some of our findings align with existing literature, others reveal more complex patterns where perceived helpfulness matters more than access alone, such as in relation to peer support. These findings offer evidence-based pathways for organisations to strengthen workforce sustainability in community-based palliative and end-of-life care services.

## Supplemental Material

sj-docx-1-pmj-10.1177_02692163251395576 – Supplemental material for Workplace support, wellbeing and intention to leave among lone working healthcare assistants providing palliative and end-of-life care in the community: A mixed methods study Supplemental material, sj-docx-1-pmj-10.1177_02692163251395576 for Workplace support, wellbeing and intention to leave among lone working healthcare assistants providing palliative and end-of-life care in the community: A mixed methods study by Katarzyna A. Patynowska, Emma Maun, Erin Raquel Fantoni, Tracey McConnell, Anne Finucane, Jonathan Clemo, Epiphany Leone, Natasha Wynne, Colette McAtamney and Felicity Hasson in Palliative Medicine

## References

[1] Etkind SN Bone AE Gomes B , et al How many people will need palliative care in 2040? Past trends, future projections and implications for services. BMC Med 2017; 15(1): 102. 28514961 10.1186/s12916-017-0860-2 PMC5436458

[2] Kane PM Daveson BA Ryan K , et al The need for palliative care in Ireland: a population-based estimate of palliative care using routine mortality data, inclusive of nonmalignant conditions. J Pain Symptom Manag 2015; 49(4): 726–733.e1. 10.1016/j.jpainsymman.2014.09.011 25461670

[3] Bone AE Gomes B Etkind SN , et al What is the impact of population ageing on the future provision of end-of-life care? Population-based projections of place of death. Palliat Med 2018; 32(2): 329–336. 29017018 10.1177/0269216317734435 PMC5788077

[4] Sleeman KE de Brito M Etkind S , et al The escalating global burden of serious health-related suffering: projections to 2060 by world regions, age groups, and health conditions. Lancet Glob Health 2019; 7(7): e883–e892. 10.1016/S2214-109X(19)30172-X PMC6560023 31129125

[5] Dhalwani NN O’Donovan G Zaccardi F , et al Long terms trends of multimorbidity and association with physical activity in older English population. Int J Behav Nutr Phys Act 2016; 13(1): 8. 26785753 10.1186/s12966-016-0330-9 PMC4717631

[6] Duffield CM Twigg DE Pugh JD , et al The use of unregulated staff: Time for Regulation? Policy Polit Nurs Pract 2014; 15(1–2): 42–48. 24705459 10.1177/1527154414529337

[7] Veras M Paquet N Oliveira EN , et al Unregulated health care workers in the care of aging populations: similarities and differences between Brazil and Canada. Fam Med Community Health 2016; 4(1): 3–14.

[8] Fitzgerald L Gathara D McKnight J , et al Are health care assistants part of the long-term solution to the nursing workforce deficit in Kenya? Hum Resour Health 2020; 18(1): 79. 33081790 10.1186/s12960-020-00523-6 PMC7576771

[9] Afzal A Stolee P Heckman GA , et al The role of unregulated care providers in Canada-a scoping review. Int J Older People Nurs 2018; 13(3): e12190. 10.1111/opn.12190 29575512

[10] Jackson J Gadimova F Epko S. A nurse by any other name? An international comparison of nomenclature and regulation of healthcare assistants. Int J Nurs Stud Adv 2024; 6: 100200. 38746790 10.1016/j.ijnsa.2024.100200 PMC11080291

[11] Fee A Muldrew D Slater P , et al The roles, responsibilities and practices of healthcare assistants in out-of-hours community palliative care: a systematic scoping review. Palliat Med 2020; 34(8): 976–988. 32538311 10.1177/0269216320929559 PMC7448826

[12] Devlin M McIlfatrick S. The role of the home-care worker in palliative and end-of-life care in the community setting: a literature review. Int J Palliat Nurs 2009; 15(11): 526–532. 20081726 10.12968/ijpn.2009.15.11.45491

[13] Herber OR Johnston BM. The role of healthcare support workers in providing palliative and end-of-life care in the community: a systematic literature review. Health Soc Care Community 2013; 21(3): 225–235. 22974295 10.1111/j.1365-2524.2012.01092.x

[14] Gershater MA Brenner J Nordberg M , et al Nurse assistants’ perception of caring for older persons who are dying in their own home: an interview study. BMC Palliat Care 2024; 23(1): 70. 38468298 10.1186/s12904-024-01399-2 PMC10929109

[15] Hasson F Slater P Fee A , et al The impact of covid-19 on out-of-hours adult hospice care: an online survey. BMC Palliat Care 2022; 21(1): 94. 35642052 10.1186/s12904-022-00985-6 PMC9155980

[16] Patynowska KA McConnell T McAtamney C , et al ‘That just doesn’t feel right at times’ – lone working practices, support and educational needs of newly employed healthcare assistants providing 24/7 palliative care in the community: a qualitative interview study. Palliat Med 2023; 37(8): 1183–1192. 37334445 10.1177/02692163231175990 PMC10503246

[17] Jansen BW Brazil K Passmore P , et al Exploring healthcare assistants’ role and experience in pain assessment and management for people with advanced dementia towards the end of life: a qualitative study. BMC Palliat Care 2017; 16(1): 6. 28103847 10.1186/s12904-017-0184-1 PMC5247820

[18] Franzosa E Tsui EK Baron S. “Who’s caring for us?”: understanding and addressing the effects of emotional labor on home health aides’ well-being. Gerontologist 2019; 59(6): 1055–1064. 30124808 10.1093/geront/gny099

[19] Saari M Xiao S Rowe A , et al The role of unregulated care providers in home care: a scoping review. J Nurs Manag 2018; 26(7): 782–794. 29708290 10.1111/jonm.12613

[20] McPherson CJ Etele J Ta VC , et al Unregulated care providers’ engagement in palliative care to older clients and their families in the home setting: a mixed methods study. BMC Palliat Care 2019; 18(1): 52. 31279338 10.1186/s12904-019-0442-5 PMC6612081

[21] Health and Safety Executive. Lone working: Protect those working alone, https://www.hse.gov.uk/lone-working/employer/index.htm (2025).

[22] Hughes P Ferrett E. Introduction to health and safety at work. Routledge, 2011.

[23] Ogoegbunam KO. Working alone: the risk of lone working with home healthcare workers and the effectiveness of safety voice in the home healthcare Industry. Master’s Thesis]. East Carolina University, 2020.

[24] Han E Kim UJ Lee Y , et al Association between lone work and self-rated health status: using the 5th Korean Working Conditions Survey. Ann Occup Environ Med 2023; 35: e29. 10.35371/aoem.2023.35.e29 PMC10493374 37701491

[25] Boerner K Burack OR Jopp DS , et al Grief after patient death: direct care staff in nursing homes and homecare. J Pain Symptom Manag 2015; 49(2): 214–222. 10.1016/j.jpainsymman.2014.05.023 PMC4300283 24996033

[26] Boerner K Gleason H Jopp DS. Burnout after patient death: challenges for direct care workers. J Pain Symptom Manag 2017; 54(3): 317–325. 10.1016/j.jpainsymman.2017.06.006 PMC5610096 28797866

[27] Gray B. The emotional labour of nursing 1: exploring the concept. Nurs Times 2009; 105(8): 26–29. 19331081

[28] Riley R Weiss MC. A qualitative thematic review: emotional labour in healthcare settings. J Adv Nurs 2016; 72(1): 6–17. 26212890 10.1111/jan.12738

[29] Lovatt M Nanton V Roberts J , et al The provision of emotional labour by health care assistants caring for dying cancer patients in the community: a qualitative study into the experiences of health care assistants and bereaved family carers. Int J Nurs Stud 2015; 52(1): 271–279. 25468132 10.1016/j.ijnurstu.2014.10.013

[30] Devlin M McIlfatrick S. Providing palliative and end-of-life care in the community: the role of the home-care worker. Int J Palliat Nurs 2010; 16(4): 195–203. 20559182 10.12968/ijpn.2010.16.4.47786

[31] Forward C Bayley Z Walker L , et al Homecare workers needs and experiences in end of life care: rapid review. BMJ Support Palliat Care 2024; 14: e2330–e2340. 10.1136/spcare-2023-004737 PMC11671894 38490719

[32] Yeh IL Samsi K Vandrevala T , et al Constituents of effective support for homecare workers providing care to people with dementia at end of life. Int J Geriatr Psychiatry 2019; 34(2): 352–359. 30430628 10.1002/gps.5027

[33] Abrams R Vandrevala T Samsi K , et al The need for flexibility when negotiating professional boundaries in the context of home care, dementia and end of life. Ageing Soc 2019; 39(9): 1976–1995.

[34] Schneider J Pollock K Wilkinson S , et al The subjective world of home care workers in dementia: an “order of worth” analysis. Home Health Care Serv Q 2019; 38(2): 96–109. 30794075 10.1080/01621424.2019.1578715

[35] Leverton M Burton A Beresford-Dent J , et al ‘You can’t just put somebody in a situation with no armour’. An ethnographic exploration of the training and support needs of homecare workers caring for people living with dementia. Dementia 2021; 20(8): 2982–3005. 34111969 10.1177/14713012211023676 PMC8678657

[36] Craftman ÅG Pakpour AH Calderon H , et al Home care assistants’ attitudes and perceptions of caring for people at the end of life in their homes in Sweden. Health Soc Care Community 2022; 30(5): e2648-e2656. DOI: 10.1111/hsc.13708. 35018690

[37] Denton MA Zeytinoğlu IU Davies S. Working in clients’ homes: the Impact on the mental health and well-being of visiting home care workers. Home Health Care Serv Q 2002; 21(1): 1–27. 10.1300/J027v21n01_01 12196932

[38] Kinman G Teoh K Harriss A. Supporting the well-being of healthcare workers during and after COVID-19. Occup Med 2020; 70(5): 294–296. 10.1093/occmed/kqaa096 PMC7313855 32428225

[39] Maben J. Peccei R Adams M , et al. Exploring the relationship between patients’ experiences of care and the influence of staff motivation, affect and wellbeing. London: National Institute for Health Research, 2012.

[40] Blake H Zhou D Batt ME. Five-year workplace wellness intervention in the NHS. Perspect Public Health 2013; 133(5): 262–271. 23771680 10.1177/1757913913489611

[41] Bamforth K Rae P Maben J , et al Perceptions of healthcare professionals’ psychological wellbeing at work and the link to patients’ experiences of care: a scoping review. Int J Nurs Stud Adv 2023; 5: 100148. 38746580 10.1016/j.ijnsa.2023.100148 PMC11080414

[42] Ravalier JM McVicar A Boichat C. Work stress in NHS employees: a mixed-methods study. Int J Environ Res Public Health 2020; 17(18): 6464. 32899897 10.3390/ijerph17186464 PMC7559167

[43] Back AL Steinhauser KE Kamal AH , et al Building resilience for palliative care clinicians: an approach to burnout prevention based on individual skills and workplace factors. J Pain Symptom Manag 2016; 52(2): 284–291. 10.1016/j.jpainsymman.2016.02.002 26921494

[44] May S Gabb F Ignatyev Y , et al Mental and physical well-being and burden in palliative care nursing: a cross-setting mixed-methods study. Int J Environ Res Public Health 2022; 19(10): 6240. 35627781 10.3390/ijerph19106240 PMC9141775

[45] Beck I Pålsson C Tops AB. Upholding an ideal image of palliative work in the face of obstacles. Int J Palliat Nurs 2018; 24(12): 611–617. 30571249 10.12968/ijpn.2018.24.12.611

[46] Findlay GM Robertson N. How do home care workers experience a client’s death, professionally and personally? A systematic review and meta-synthesis. OMEGA - J Death Dying 2024; 0(0). DOI: 10.1177/00302228241302431. 10.1177/00302228241302431 PMC13646400 39608766

[47] Creswell JW Clark VLP . Designing and conducting mixed methods research. Sage Publications, 2017.

[48] O’cathain A Murphy E Nicholl J. The quality of mixed methods studies in Health Services Research. J Health Serv Res Policy 2008; 13(2): 92–98. 10.1258/jhsrp.2007.007074 18416914

[49] Stewart-Brown S Tennant A Tennant R , et al Internal construct validity of the Warwick-Edinburgh Mental Well-Being Scale (WEMWBS): a Rasch analysis using data from the Scottish Health Education Population Survey. Health Qual Life Outcomes 2009; 7(1): 15. 19228398 10.1186/1477-7525-7-15 PMC2669062

[50] Tennant R Hiller L Fishwick R , et al The Warwick-Edinburgh Mental Well-Being Scale (WEMWBS): development and UK validation. Health Qual Life Outcomes 2007; 5(1): 63. 18042300 10.1186/1477-7525-5-63 PMC2222612

[51] Ng Fat L Scholes S Boniface S , et al Evaluating and establishing national norms for mental wellbeing using the short Warwick-Edinburgh Mental Well-Being Scale (SWEMWBS): findings from the Health Survey for England. Qual Life Res 2017; 26: 1129–1144. 27853963 10.1007/s11136-016-1454-8 PMC5376387

[52] The University of Warwick. Collect, score, analyse and interpret WEMWBS. The University of Warwick, 2023.

[53] Bothma CFC Roodt G . The validation of the turnover intention scale. SA Journal of Human Resource Management 2013; 11(1): 1–12.

[54] Roodt G. Turnover intentions. Unpublished document. University of Johannesburg, 2004.

[55] Hood C Patton R. Exploring the role of psychological need fulfilment on stress, job satisfaction and turnover intention in support staff working in inpatient mental health hospitals in the NHS: a self-determination theory perspective. J Ment Health 2022; 31(5): 692–698. 34565267 10.1080/09638237.2021.1979487

[56] Tan SL Zhou H Thian HJ , et al Influence of perceived job demands on professional quality of life and turnover intentions of Haematology Nurses: a cross-sectional study. J Nurs Manag 2024; 2024: 1–6626516. 10.1155/2024/6626516 PMC11919190 40224820

[57] Marie Curie . Resilience based clinical supervision policy. Marie Curie, 2023.

[58] Stacey G Aubeeluck A Cook G , et al. A case study exploring the experience of resilience-based clinical supervision and its influence on care towards self and others among student nurses. Int Pract Develop J 2017; 7(2): 1–16.

[59] Marie Curie . Health and wellbeing hub. Marie Curie, 2023.

[60] The Point of Care Foundation. About Schwartz Rounds. The Point of Care Foundation, 2024.

[61] Braun V Clarke V. Using thematic analysis in psychology. Qual Res Psychol 2006; 3(2): 77–101.

[62] Gale NK Heath G Cameron E , et al Using the framework method for the analysis of qualitative data in multi-disciplinary health research. BMC Med Res Methodol 2013; 13(1): 117. 24047204 10.1186/1471-2288-13-117 PMC3848812

[63] Green J Willis K Hughes E , et al Generating best evidence from qualitative research: the role of data analysis. Aust N Z J Public Health 2007; 31(6): 545–550. 18081575 10.1111/j.1753-6405.2007.00141.x

[64] Miles MB Huberman AM Saldana J. Fundamentals of qualitative data analysis in qualitative data analysis: a methods sourcebook. Sage Publication, 2014, pp.69–104.

[65] Moran-Ellis J Alexander VD Cronin A , et al Triangulation and integration: processes, claims and implications. Qual Res 2006; 6(1): 45–59.

[66] Murphy E Dingwall R Greatbatch D , et al Qualitative research methods in health technology assessment: a review of the literature. Health Technol Assess 1998; 2(16): iii–ix, 1–274. 9919458

[67] Wiltshire G Ronkainen N. A realist approach to thematic analysis: making sense of qualitative data through experiential, inferential and dispositional themes. J Crit Realism 2021; 20(2): 159–180.

[68] Berends L Johnston J. Using multiple coders to enhance qualitative analysis: the case of interviews with consumers of drug treatment. Addict Res Theory 2005; 13(4): 373–381.

[69] Birt L Scott S Cavers D , et al Member checking: a tool to enhance trustworthiness or merely a nod to validation? Qual Health Res 2016; 26(13): 1802–1811. 27340178 10.1177/1049732316654870

[70] Mabry L Parker KN Thompson SV , et al Protecting workers in the home care industry: workers’ experienced job demands, resource gaps, and benefits following a socially supportive intervention. Home Health Care Serv Q 2018; 37(3): 259–276. 29718782 10.1080/01621424.2018.1470590

[71] Poon A Luebke M Loughman J , et al Computer-mediated sharing circles for intersectional peer support with home care workers. Proc ACM Hum Comput Interact 2023; 7(CSCW1): 1–35.

[72] De Kock JH Ann Latham H Cowden RG , et al The mental health of NHS staff during the COVID-19 pandemic: two-wave Scottish cohort study. BJPsych Open 2022; 8(1): e23. 10.1192/bjo.2021.1079 PMC8755549 35043077

[73] Finucane AM Hulbert-Williams NJ Swash B , et al Feasibility of RESTORE: an online acceptance and commitment therapy intervention to improve palliative care staff wellbeing. Palliat Med 2023; 37(2): 244–256. 36576308 10.1177/02692163221143817 PMC13033365

[74] Hynes J Maffoni M Argentero P , et al Palliative medicine physicians: doomed to burn? BMJ Support Palliat Care 2019; 9(1): 45–46. 10.1136/bmjspcare-2018-001731 30782969

[75] Dijxhoorn AQ Brom L van der Linden YM , et al Prevalence of burnout in healthcare professionals providing palliative care and the effect of interventions to reduce symptoms: a systematic literature review. Palliat Med 2021; 35(1): 6–26. 33063609 10.1177/0269216320956825

[76] Peters E. Compassion fatigue in nursing: a concept analysis. Wiley Online Library, 2018, pp.466–480. 10.1111/nuf.12274 29962010

[77] Portoghese I Galletta M Larkin P , et al Compassion fatigue, watching patients suffering and emotional display rules among hospice professionals: a daily diary study. BMC Palliat Care 2020; 19(1): 23. 32098618 10.1186/s12904-020-0531-5 PMC7043034

[78] Papworth A Ziegler L Beresford B , et al Psychological well-being of hospice staff: systematic review. BMJ Support Palliat Care 2023; 13(e3): e597–e611. 10.1136/spcare-2022-004012 37098444

[79] McConnell T Porter S. The experience of providing end of life care at a children’s hospice: a qualitative study. BMC Palliat Care 2017; 16(1): 15. 28193270 10.1186/s12904-017-0189-9 PMC5307784

[80] Swetz KM Harrington SE Matsuyama RK , et al Strategies for avoiding burnout in hospice and palliative medicine: peer advice for physicians on achieving longevity and fulfillment. J Palliat Med 2009; 12(9): 773–777. 19622012 10.1089/jpm.2009.0050

[81] Mills J Wand T Fraser JA. Exploring the meaning and practice of self-care among palliative care nurses and doctors: a qualitative study. BMC Palliat Care 2018; 17(1): 63. 29669559 10.1186/s12904-018-0318-0 PMC5907186

[82] Edmonds KP Yeung HN Onderdonk C , et al Clinical supervision in the palliative care team setting: a concrete approach to team wellness. J Palliat Med 2015; 18(3): 274–277. 25517027 10.1089/jpm.2014.0248

[83] Stacey G Aubeeluck A Cook G , et al A case study exploring the experience of resilience-based clinical supervision and its influence on care towards self and others among student nurses. Int Pract Dev J 2017; 7(2): 1–16.

[84] Cutcliffe JR Sloan G Bashaw M. A systematic review of clinical supervision evaluation studies in nursing. Int J Ment Health Nurs 2018; 27(5): 1344–1363. 29446513 10.1111/inm.12443

[85] Keane S Ryan A Adams N , et al Palliative care nurses’ experiences of clinical supervision: a qualitative evidence synthesis. Int J Palliat Nurs 2020; 26(8): 413–423. 33331208 10.12968/ijpn.2020.26.8.413

[86] Chan ZC Tam WS Lung MK , et al A systematic literature review of nurse shortage and the intention to leave. J Nurs Manag 2013; 21(4): 605–613. 23406374 10.1111/j.1365-2834.2012.01437.x

[87] Ligibel JA Goularte N Berliner JI , et al Well-being parameters and intention to leave current institution among academic physicians. JAMA Netw Open 2023; 6(12): e2347894. 10.1001/jamanetworkopen.2023.47894 PMC10724765 38100103

[88] Zaheer S Ginsburg L Wong HJ , et al Turnover intention of hospital staff in Ontario, Canada: exploring the role of frontline supervisors, teamwork, and mindful organizing. Hum Resour Health 2019; 17(1): 66. 31412871 10.1186/s12960-019-0404-2 PMC6693251

[89] Hayes LJ O’Brien-Pallas L Duffield C , et al Nurse turnover: a literature review – an update. Int J Nurs Stud 2012; 49(7): 887–905. 22019402 10.1016/j.ijnurstu.2011.10.001

[90] Department of Health and Social Care. Adult social care workforce survey: April 2025 report [Internet]. England: Department of Health and Social Care. https://www.gov.uk/government/statistics/adult-social-care-workforce-survey-april-2025/adult-social-care-workforce-survey-april-2025-report

