# Supplementary material for: Workplace support, wellbeing and intention to leave among lone working healthcare assistants providing palliative and end-of-life care in the community: A mixed methods study
Source: Palliat Med. 2025 Dec 6;40(2):194–205. doi: 10.1177/02692163251395576 (PMC12909606; doi:10.1177/02692163251395576)
Supplement: sj-docx-1-pmj-10.1177_02692163251395576 – Supplemental material for Workplace support, wellbeing and intention to leave among lone working healthcare assistants providing palliative and end-of-life care in the community: A mixed methods study [file sj-docx-1-pmj-10.1177_02692163251395576.docx]

**Supplemental material for paper: ‘Workplace support, wellbeing and intention to leave among lone working Healthcare Assistants providing palliative and end-of-life care in the community: A mixed methods study’**

Contents

[Questionnaire 1](#_Toc195086804)

[Interview schedule 10](#_Toc195086805)

[Results 12](#_Toc195086806)

# **Questionnaire**

Dear All

Before you begin, please click the following link which will redirect you to the Participant Information Sheet. Please take the time to read the Participant Information Sheet which is available via the link below.

<<LINK>>

This survey is for Healthcare Assistants who spend at least part of their work hours lone working. By lone working, we mean visiting patients in their homes on your own, without any colleagues physically present to assist you.

The survey will take approximately **10 minutes** to complete, and your participation is voluntary. By completing this survey, you are consenting to participate in this study.

All data will be anonymous and will remain confidential.

For further information, do not hesitate to contact: [kasia.patynowska@mariecurie.org.uk](mailto:kasia.patynowska@mariecurie.org.uk)

**Screening Question**

I confirm that I am a Healthcare Assistant and I spend at least part of my work-hours lone-working: *(By lone working, we mean visiting patients in their homes on your own, without any colleagues physically present to assist you).*

Yes/No

**Wellbeing Scale**

For more information please refer to [The Warwick-Edinburgh Mental Wellbeing Scale (WEMWBS)](https://warwick.ac.uk/fac/sci/med/research/platform/wemwbs/)

**Current Support**

You will now be asked a series of questions about forms of support you may currently use. For any forms of support you do use, you’ll be given a follow-up question to express how this support has been helpful.

*Researcher note: For legibility, the types of questions have been presented in separate tables below, but in the MS Forms, participants will be shown the respective “helpfulness” question immediately after indicating they have used a support. Those who have not used a support will skip its “helpfulness” question and be asked if they have received another form of support.*

*Initial questions on awareness and usage*

|  | I have never heard of this support/I do not know if this support is available to me or not | This support is not available to me | This support is available, but I have not used it. | This support is available, and I have used it. |
| --- | --- | --- | --- | --- |
| Contact with other HCAs, such as texts, phone calls, or meetings in person | 0 | 1 | 2 | 3 |
| Contact with other practitioners e.g. Registered Nurses | 0 | 1 | 2 | 3 |
| Contact with line manager | 0 | 1 | 2 | 3 |
| Support from my family and/or friends about work-based concerns | 0 | 1 | 2 | 3 |
| Employee Assistance Programme | 0 | 1 | 2 | 3 |
| Online Health & Wellbeing Hub | 0 | 1 | 2 | 3 |
| Unmind app | 0 | 1 | 2 | 3 |
| Clinical Supervision | 0 | 1 | 2 | 3 |
| Swartz Rounds | 0 | 1 | 2 | 3 |
| Others: open | 0 | 1 | 2 | 3 |

If you selected “other” as a type of support, please describe it here. You can describe more than one other type of support if needed : ___________[OPEN]

*Questions on helpfulness of support*

|  | Not at all helpful | A little helpful | Moderately helpful | Very helpful |
| --- | --- | --- | --- | --- |
| Contact with other HCAs, such as texts, phone calls, or meetings in person | 1 | 2 | 3 | 4 |
| Contact with other practitioners e.g. Registered Nurses | 1 | 2 | 3 | 4 |
| Contact with line manager | 1 | 2 | 3 | 4 |
| Support from my own family about work-based concerns | 1 | 2 | 3 | 4 |
| Support from friends outside of work | 1 | 2 | 3 | 4 |
| Employee Assistance Programme | 1 | 2 | 3 | 4 |
| Online Health & Wellbeing Hub | 1 | 2 | 3 | 4 |
| Unmind app | 1 | 2 | 3 | 4 |
| Clinical Supervision | 1 | 2 | 3 | 4 |
| Swartz Rounds | 1 | 2 | 3 | 4 |
| Others: open | 1 | 2 | 3 | 4 |

**Intentions to Leave or Stay in Role**

Intention to leave was assessed using the six-item Turnover Intention Scale (TIS_6)(1, 2). Please refer to the original papers for more information:

1. Bothma CFC, Roodt, G. The validation of the turnover intention scale : original research. SA Journal of Human Resource Management. 2013;11(1):1-12.

2. Roodt G. Turnover intentions. Unpublished document. Johannesburg, South Africa: University of Johannesburg. 2004.

**Open Questions**

What factors come to mind when you think of reasons *not to stay* in your current role? ____________________ [OPEN]

What factors come to mind when you think of reasons *to stay* in your current role?

____________________ [OPEN]

And finally, what do you think the organisation could do to support Health Care Assistants and help them stay in their role? I.e. increase retention?

____________________ [OPEN]

**Demographic/workplace questionnaire**

Thank you for taking the time to respond to this survey. The questions in this final section will help us understand who is represented in our sample and who our findings best apply to.

**You can select "prefer not to say" or skip questions, if you wish.**

*Please tick one answer.*

### 1a. How would you describe your gender identity? (If you would rather not share your answer, please select the “Prefer not to say” option)

### Male

### Female

### Non-binary

### Prefer not to say

### Prefer to self-describe _______

2. What is your year of birth? …………….

3. Ethnicity: The next questions are about your ethnicity. As people can come from multiple ethnicities, you will be asked multiple questions.

*Researcher note. In the MS Form, participants will be given yes/no response options to if they have an Asian/Black/White/Other ethnic identity. When they answer no, the form logic will take them to the next identity group. When they answer yes, the form logic will take them to a follow-up question that lists all of the categories within an identity group based on the UK census, as well as an “prefer to self-describe” open option.*

*In lieu of the census’s approach of a separate category for people of mixed ethnic background, the survey will allow participants to select as many identities as may apply to them.*

What ethnic groups do you associate with? Please select any/all groups you identify with, then you’ll be given the opportunity to provide more details if you would like to do so:

Do you have an Asian ethnicity? Yes  No

Please select any of your specific Asian identities:

Indian

Pakistani

Bangladeshi

Chinese

British

Self-describe: ____________ [OPEN]

Prefer not to say

Do you have a Black ethnicity? Yes  No

Please select any of your specific Black identities:

African

Caribbean

British

Self-describe: ____________ [OPEN]

Prefer not to say

Do you have a White ethnicity? Yes  No

Please select any of your specific White identities:

English

Irish

Northern Irish

Scottish

Welsh

British

Gypsy or Irish Traveller

Roma

Continental Europe

Self-describe: ____________ [OPEN]

Prefer not to say

Do you have any other ethnicities? Yes  No

Please select any of your other ethnicities. If you need to add more than one, you can list multiple ethnicities in the "other" box.

Arab

Self-describe: ____________ [OPEN]

Prefer not to say

### 4. Do you work ……? Contracted hours

### Bank/As and when hours

5. Do you work…….? Full-time

Part Time

6. Which place do you work in?

Northern Ireland

Scotland North/West

Scotland South/East

Wales

North East & Yorkshire

North West

West Midlands

East of England

London

South West

South East

Prefer not to say

8.What type of service do you spend the most time working in?

Planned variable (e.g. overnight visits)

Multi-visit

Rapid Response

Other ­­­_________

9. Do you also work in any other type of service? Tick all that apply.

Planned variable (e.g. overnight visits)

Multi-visit

Rapid Response

NHS

Private sector

NA: I don’t work in any other types of services

Other ­­­_________

10. What proportion of your role is spent lone working?

None/almost none

Some

About half

Most

All/almost all

11. How long, in years, have you worked as a Healthcare Assistant in Community Service in this organisation?

______ [OPEN]

12. How long, in years, had you worked as a Healthcare Assistant before joining current organisation? If you have no prior experience in this role, please list NA.

______ [OPEN]

13. If you have any other experience working in health and care setting, can you please describe briefly below?

______ [OPEN]

**Thank you for completing the questionnaire!**

If you’d like to take part in the draw for £30 gift vouchers and/or register your interest to take part in the interview for the same study, please click on the link below.

Information you can provide below will not be linked to this questionnaire in any way.

<<LINK>>

# **Interview schedule**

Opening Statements

The researcher will start with an opening statement as follows:

- *Thank you for taking time for this interview today, it* will last between 30-60 minutes *and can be stopped at any time*.
- *I want to remind you that there are no right, or wrong answers. You are asked not to disclose any specific patient cases or identifying features in your responses. As per the PIS/Consent form all* *disclosed information will be treated confidentially unless required by law, i.e. there is a risk to yourself or others.*
- *Our questions today have been informed by the survey that was sent out to all community based lone working HCAs in Marie Curie, in terms of what issues to focus on. Please keep in mind that all the questions are focusing on your perspective of HCAs work-related psychological wellbeing, support needs and staff retention.*

The researcher will seek verbal consent for digital recording (video and audio), otherwise notes will be taken

Opening questions

- Can you outline how long you have been working as a Healthcare Assistants in Marie Curie Community Service?
- What is your previous work experience?
- In what services do you usually work and what hours do you do?
- How much lone working do you do in a typical month?
- How much do you work within clinical working hours (8am-6pm) and during the out-of-hours working period (6pm-8am)?

1. Thinking about your role as HCA, can you describe your **work-life balance**? How does your as HCA impact your work-life balance? Does it have any impact on your intention to stay, or leave their job? In what way?
2. What type of **training, development and career progression opportunities** in Marie Curie are available to you? Does it have any impact on your work-related wellbeing? In what way? Do you think these also impact on your intention to stay, or leave their job? In what way?
3. How important is **feeling valued** to you in your role in terms of your psychological wellbeing at work? Could you tell me a little more about why you think that? In what way does it impact on your intention to stay, or leave their job?
4. What does **working as HCA mean to you**? Do you think your role as a HCA has a positive **impact on the lives** of patients/families? In what way? What impact does it have on your wellbeing at work? What impact does it have on your intention to stay in/leave your job?
5. How **contact with other HCAs, healthcare practitioners and line manager** impact on your wellbeing? How would you describe the support from these sources? How that support looks like and what does it incorporate? What impact do these have on your intention to stay in/leave your job?
6. What is **the role of line manager in the support** offered to HCAs? *(explore the line manager’s role in terms of providing the support, facilitating support e.g. team meetings and signposting to support)*
7. Very few people who completed the survey heard and/or used **Employee Assistance Programme, Wellbeing Hub, Schwartz rounds** – have you heard of these? What do you think are the reasons for low awareness and usage of those support systems? *(explore differences between formal vs. informal or person to person vs. virtual support)*
8. How do you think people **choose the sources of support** they access? *(explore if there flexibility in accessing various support systems or people tend to use the same sources; are people use various sources of support for ‘wellbeing at work’ vs. ‘wellbeing as a person’)*
9. What support has been **most and least helpful to you** at work? In what situations did you use it? *(explore appropriateness of support accessed for specific issues, i.e. was support accessed the most appropriate in a specific situations)*
10. **Have you considered leaving** your current role? Can you tell me more about that, please? *(explore the main considerations for staying and leaving)*
11. What **support intervention do you think has the most potential** to help improve the wellbeing, job satisfaction and retention of HCAs? Why did you choose that particular one?
12. What would your **second choice** be? Why did you choose that particular one? What would be your second choice of the intervention?

Final question

Is there something else you would like to add before we finish?

End of interview

Thank you very much for your time and for sharing your thoughts

Signpost to PIS support section

# **Results**

Table S1: Reliability and validity analysis for wellbeing and intention to leave indicators.

| Measure | | Wellbeing | Intention to leave |
| --- | --- | --- | --- |
| Reliability | Cronbach’s alpha, *α* | 0.92 | 0.76 |
|  |  |  |  |
|  | Inter-item correlation, *M* | 0.46 | 0.34 |
|  |  |  |  |
| Validity | Confirmatory factor analysis |  |  |
|  | Χ^2^ | 271.76, *p* < .001^1^ | 25.23, *p* = .003^2^ |
|  | CFI | .88 | .96 |
|  | RMSEA | .108 | .09 |
|  | SRMR | .06 | .05 |

Notes: ^1^ Χ^2^ (df:77), ^2^ Χ^2^ (df:9)

Table S2: Comparison of population characteristics (lone working population healthcare assistants at 1 May 2023) and the sample

|  |  | Survey participants, N=218 | | | Population characteristics lone working healthcare assistants, N=969 | | |
| --- | --- | --- | --- | --- | --- | --- | --- |
|  |  | Frequency | % of valid responses / mean(SD) | Range | Frequency | % of valid responses / mean(SD) | Range |
| Gender | Female | 202 | 92.7 |  | 905 | 94.9 |  |
|  | Male | 9 | 4.1 |  | 49 | 5.1 |  |
|  |  |  |  |  |  |  |  |
| Age | Mean (SD) | 200 | 51.3 (11.0) | 23-72 years | 969 | 50.3 (11.4) | 20-77 years |
|  |  |  |  |  |  |  |  |
| Ethnicity | White | 181 | 85.0 |  | 730 | 89.4 |  |
|  | Ethnic minorities | 32 | 15.0 |  | 87 | 10.6 |  |
|  |  |  |  |  |  |  |  |
|  | Asian | 3 | 1.4 |  | 15 | 1.8 |  |
|  | Black | 23 | 10.8 |  | 59 | 7.2 |  |
|  | Other | 6 | 2.8 |  | 13 | 1.6 |  |
|  |  |  |  |  |  |  |  |
| Type of hours | Full time | 83 | 39.3 |  | 114 | 11.8 |  |
|  | Part time (including variable hours) | 128 | 60.7 |  | 854 | 88.2 |  |
|  |  |  |  |  |  |  |  |
| Place of work | England | 144 | 68.6 |  | 640 | 66.0 |  |
|  | Northern Ireland | 18 | 8.6 |  | 85 | 8.8 |  |
|  | Scotland | 39 | 18.6 |  | 148 | 15.3 |  |
|  | Wales | 9 | 4.3 |  | 96 | 9.9 |  |

Notes: missing data for the population of lone working HCAs, Gender (15, 1.5%, recorded as ‘Not provided’), Ethnicity (152, 15.7%, ‘Not provided’), Hours (1, 0.1%).

Table S3: Correlation of wellbeing and intention to leave with demographic and work characteristics

|  | N | Correlation (p value) with wellbeing | Correlation (p value) with intention to leave |
| --- | --- | --- | --- |
| Time Spent Lone Working | 218 | -.03 (.68) | -.11 (.10) |
| Contract or Bank Hours | 217 | .12 (.08) | -.06 (.37) |
| Full- or Part-time Hours | 211 | .03 (.64) | -.05 (.46) |
| Age | 200 | .03 (.65) | -.08 (.24) |
| Total Years of HCA Experience | 199 | .06 (.44) | .10 (.16) |

Notes: Spearman correlations

Table S4: Participant awareness and accessing of different forms of support: N, % and wellbeing and intention to leave mean scores (SD)

|  |  | Aware of support | Accessed Support | Wellbeing and intention to leave scores for participants who accessed support,  mean (SD) | |
| --- | --- | --- | --- | --- | --- |
|  |  | N (%) | N (%) | Wellbeing,  (range 14-70) | Intention to leave, (range 6-30) |
| Support from known persons | Y | 218 (100) | 212 (97.2) | |  |
|  | N | 0 | 6 (2.8) |  |  |
|  |  |  |  |  |  |
| Line manager | Y | 215 (98.6) | 190 (87.2) | 53.1 (8.3) | 14.3 (5.1) |
|  | N | 3 (1.4) | 28 (12.8) | 45.5 (8.0) | 18.6 (4.6) |
|  |  |  |  |  |  |
| Clinical supervision | Y | 213 (97.7) | 166 (76.2) | 52.3 (8.5) | 14.9 (5.1) |
|  | N | 5 (2.3) | 52 (23.8) | 51.7 (9.0) | 14.6 (5.8) |
|  |  |  |  |  |  |
| Family and friends | Y | 211 (96.8) | 121 (55.5) | 52.4 (8.3) | 14.9 (5.4) |
|  | N | 7 (3.2) | 97 (44.5) | 51.8 (9.1) | 14.8 (5.2) |
|  |  |  |  |  |  |
| Other healthcare assistants | Y | 205 (94.0) | 157 (72.0) | 52.7 (8.5) | 15.6 (5.3) |
|  | N | 13 (6.0) | 61 (28) | 50.8 (8.9) | 14.6 (5.2) |
|  |  |  |  |  |  |
| Other healthcare workers | Y | 198 (90.8) | 149 (68.3) | 52.8 (8.4) | 15.6 (5.1) |
|  | N | 20 (9.2) | 69 (31.7) | 50.8 (9.1) | 14.5 (5.3) |
|  |  |  |  |  |  |
| Anonymous and online self-directed support | Y | 202 (92.7) | 38 (17.4) |  |  |
|  | N | 16 (7.3) | 180 (82.6) |  |  |
|  |  |  |  |  |  |
| Health and Wellbeing Hub | Y | 188 (86.2) | 19 (8.7) | 54.4 (10.6) | 13.8 (6.5) |
|  | N | 30 (13.8) | 199 (91.3) | 51.9 (8.4) | 14.9 (5.1) |
|  |  |  |  |  |  |
| Employee Assistance Program | Y | 145 (66.5) | 21 (9.6) | 54.5 (11.1) | 14.5 (5.2) |
|  | N | 73 (33.5) | 197 (90.4) | 51.9 (8.3) | 14.9 (5.3) |
|  |  |  |  |  |  |
| Unmind app | Y | 78 (35.8) | 7 (3.2) | 51.6 (9.1) | 13.6 (5.0) |
|  | N | 140 (64.2) | 211 (96.8) | 52.2 (8.6) | 14.9 (5.3) |
|  |  |  |  |  |  |
| Schwartz Rounds | Y | 53 (24.3) | 4 (1.8) | 53.0 (7.5) | 14.0 (3.8) |
|  | N | 165 (24.3) | 214 (98.2) | 52.1 (8.7) | 14.9 (5.3) |

Notes: SD standard deviation

Table S5: Participant helpfulness of different forms of accessed support, N, % and correlations with wellbeing and intention to leave

| Form of Support | Helpfulness of support accessed (range 1-4) ^3^ | | Correlation (p value) between helpfulness of accessed support and | |
| --- | --- | --- | --- | --- |
|  | N | Mean (SD) | Wellbeing^2^ | Intention to leave^2^ |
| Support from known persons  Line manager | 190 | 3.7 (.5) | .30 (<.001) | -.22 (.002) |
| Clinical supervision | 166 | 3.5 (.7) | .39 (<.001) | -.32 (<.001) |
| Family and friends | 121 | 3.6 (.6) | .21 (.02) | -.12 (.17) |
| Other healthcare assistants | 157 | 3.8 (.5) | .22 (.006) | -.03 (.70) |
| Other healthcare workers  Anonymous and online self-directed support | 149 | 3.6 (.6) | .45 (<.001) | -.32 (<.001) |
| Health and Wellbeing Hub | 19 | 3.7 (.6) | .36 (.13) | -.23 (.35) |
| Employee Assistance Program | 21 | 3.6 (.8) | .20 (.38) | -.13 (.57) |
| Unmind app | 7 | 3.5 (.6) | - | - |
| Swartz rounds | 4 | 3.5 (.6) | - | - |

Notes: ^1^percentage of sample of 218 participants; SD: standard deviation; ^2^Spearman correlations presented only for those variables with more than 10 cases; ^3^response options, 1 “Not at all helpful” to 4 “Very helpful”.
